# Modified QuEChERS methodology for the analysis of OCs and PCBs in liver and its application in wild birds (white stork, red kite, and griffon vulture)

**DOI:** 10.3389/fvets.2026.1843311

**Published:** 2026-06-12

**Authors:** Yolanda Ibáñez-Pernía, Marcos Pérez-López, David Fernández-Casado, Francisco Soler-Rodríguez

**Affiliations:** Área de Toxicología, Departamento de Sanidad Animal, Facultad de Veterinaria, Universidad de Extremadura, Cáceres, Spain

**Keywords:** birds, GC–MS, liver, OCS, One Health, PCBs, POPs, QuEChERS

## Abstract

This study aimed to adapt and validate the QuEChERS method for extracting and purifying 19 persistent organic pollutants—11 organochlorine compounds and 8 polychlorinated biphenyls—for GC–MS (CNI mode) analysis in bird liver samples. After extraction with acetonitrile and salt phase separation, the extract was reconstituted in hexane and cleaned using sulfuric acid. Linearity was ≥0.9, and precision was below 20% (ranging from 0.63 to 19.35%) across concentrations of 1–100 ng/g. Limits of detection ranged from 0.20 to 2.03 ng/g for organochlorine compounds and from 0.93 to 4.49 ng/g for polychlorinated biphenyls. Limits of quantification ranged from 0.67 to 6.78 ng/g for organochlorine compounds and from 3.11 to 14.97 ng/g for polychlorinated biphenyls. The matrix effect was slightly negative for *α*-HCH, heptachlor, and aldrin. The method was applied to 60 liver samples from wild birds representing different trophic groups: 20 white storks (*Ciconia ciconia*), 20 red kites (*Milvus milvus*), and 20 griffon vultures (*Gyps fulvus*), all found dead in the wild. On average, 11 compounds were detected per sample. Results showed a wide dispersion, with extreme values, so the median was used as a more reliable measure. DDE was the most prevalent, detected in 100% of samples. Seven compounds were not detected in more than 50% of the samples. The red kite showed the highest median concentrations for all compounds except for *β*-HCH (highest in griffon vultures), DDD (highest in white storks), and PCB28 (highest in griffon vultures). Aldrin had the lowest detection frequency. Statistically significant differences were only observed for DDE, DDD, PCB153, PCB138, and PCB180. Greater persistent organic pollutants accumulation was noted in raptors.

## 1 Introduction

Persistent organic pollutants (POPs) are synthetic organic compounds that include polychlorinated biphenyls (PCBs), organochlorine pesticides (OCs), and industrial by-products such as dioxins and furans. These compounds were widely used in agriculture and industry during the mid-20th century (1). Their applications can be broadly classified into plant protection products, industrial chemicals, and unintentionally generated by-products of combustion processes.

POPs are characterized by their high resistance to degradation and their persistence in the environment for extended periods. They accumulate in the fatty tissues of organisms (bioaccumulation) (2–5) and increase in concentration along trophic levels (biomagnification) (2, 6–8). In addition, their semi-volatile nature enables long-range atmospheric transport (high temperatures promote their volatilization), allowing them to reach remote regions and pose risks to wildlife and human health (8–13).

Due to these concerns, the Stockholm Convention was adopted in 2001 to restrict and eliminate the production and use of POPs. This framework has been implemented at international and regional levels (14, 15, 83, 87), leading to progressive restrictions on these compounds.

Within this framework, given that pollutants are recognized as major drivers of biodiversity loss (86), assessing their ecological impacts is essential, with wildlife serving as indicators of environmental exposure. Global wildlife populations have declined by 60% since 1974 (90), largely due to human activities, including chemical contamination (16, 88). POPs are widely studied in wildlife and are associated with population declines and abnormalities (17). Although regulations have reduced their levels (18–23), concentrations remain high, particularly in top predators, with PCBs still among the most prevalent compounds (94), highlighting the continued need for POP monitoring in wildlife. Despite their slow metabolism, their accumulation exceeds the rates of environmental degradation, making organism concentrations reliable indicators of ecological bioavailability.

Wildlife biomonitoring is a key tool for the early detection of ecological and human health risks (86). Birds, particularly raptors, are widely used as sentinel species in biomonitoring programs (24–28), allowing the assessment of spatiotemporal trends, associated effects, and the effectiveness of regulatory measures. This approach aligns with the One Health framework, linking environmental, wildlife, and human health.

Despite their relevance for environmental monitoring, the analysis of POPs in complex biological matrices, such as liver, nervous, and adipose tissues, remains challenging due to their high lipid content, which complicates clean-up and leads to analytical interferences. Extraction is typically performed using organic solvents, followed by labor-intensive purification techniques such as liquid–liquid extraction, solid-phase extraction, gel permeation chromatography, or sulfuric acid treatment (29, 30).

To address these analytical challenges, the QuEChERS method has emerged in recent years as a widely used alternative. Developed by Anastassiades et al. (31), it enables rapid, simple, and cost-effective extraction and clean-up of a wide range of pesticides in fruits and vegetables. The acronym stands for Quick, Easy, Cheap, Effective, Rugged, and Safe, reflecting its main advantages. It is a multiresidue analytical technique with low requirements in terms of labor, time, and materials. It combines a basic extraction with acetonitrile (ACN) from homogenized samples, followed by liquid–liquid partitioning using salts such as anhydrous magnesium sulfate (MgSO₄) and sodium chloride (NaCl). The extract is then dried and cleaned using dispersive solid-phase extraction (dSPE) with MgSO₄ and sorbents such as primary secondary amine (PSA), C18, or graphitized carbon to remove interfering compounds, including organic acids, polar pigments, and sugars. After mixing and centrifugation, the extract is ready for chromatographic analysis (GC–MS or LC–MS), achieving optimal performance when triple quadrupole MS/MS detectors are used, as they provide high sensitivity without the need for extensive clean-up. However, this type of instrumentation is not available in all analytical laboratories due to its high cost.

Due to its advantages, numerous QuEChERS-based approaches have been developed for different matrices and analytes in food, environmental, and forensic toxicology (human and veterinary). Blood is the most commonly analyzed matrix, as it can be obtained from both live and dead animals. Rial-Berriel et al. (16) identified 306 toxic compounds, including 40 POPs, in raptor blood, and up to 353 compounds in camel and human serum (32). The method has also been applied to other matrices, including human breast milk and liver (33, 91, 95). Human and veterinary toxicology are closely linked, as matrices and target compounds overlap. It has also been applied to animal-derived food products and lipid-rich plant matrices (3, 92, 89), and has expanded beyond food toxicology.

However, the application of QuEChERS to complex, high-lipid matrices such as liver still requires specific adaptations to minimize co-extracted matrix components and analytical interferences (34, 35). This limits its analytical performance and often necessitates the use of advanced instrumentation such as MS/MS, which is not always available in many laboratories. Therefore, there is still a need to develop adapted, efficient, and accessible methods for the analysis of POPs in these matrices without requiring advanced instrumentation.

In veterinary forensic toxicology, the liver is the preferred organ for the post-mortem detection of toxic substances (35, 36, 93). Given that POP analysis in liver remains an important research area, the aim of this study was to adapt the QuEChERS method for the extraction and purification of OCs and PCBs in avian liver samples, followed by their analysis using GC–MS. This approach is intended to be applicable in laboratories without MS/MS instrumentation, such as those in developing countries or with limited economic resources.

The applicability of the method was evaluated by analyzing OC and PCB concentrations in 60 liver samples from birds representing three trophic levels.

## 2 Materials and methods

### 2.1 Solvents and reagents

The solvents used were acetonitrile (ACN) (Carlo Erba, residue analysis grade), tetrahydrofuran (THF) (Scharlab, HPLC grade), acetone (Scharlab, residue analysis grade), petroleum ether 40–60 °C (Scharlab, residue analysis grade), n-hexane (Scharlab, residue analysis grade), and isooctane (Scharlab, residue analysis grade).

The salts used included anhydrous MgSO₄ (Scharlab, >98%), NaCl (Scharlab, residue analysis grade), anhydrous Na₂SO₄ (Scharlab, residue analysis grade), disodium citrate (Sigma, analytical grade, >99%), and trisodium citrate dihydrate (Sigma, analytical grade, >99%).

Sulfuric acid (H₂SO₄) (Scharlab, 95%–97%, reagent grade) was also used.

### 2.2 Standards

Certified chlorinated standards of pesticides were acquired from Supelco (EPA 625/CLP Pesticide Mix containing Aldrin, *α*HCH, *β*HCH, lindane, δHCH, 4,4′-DDT, dieldrin, α-endosulfan, β-endosulfan, endosulfan sulfate, endrin, endrin aldehyde, heptachlor, and heptachlor epoxide at 2000 μg/mL in hexane:toluene (50:50); Chlordane (isomer mixture, 1,000 μg/mL in isooctane); and solid individual standards of 4,4′-DDE and 4,4′-DDD. Certified standards of PCBs at 10 ng/μl in isooctane were from Dr. Ehrenstorfer (PCB-Mix 3 containing PCB28, PCB52, PCB101, PCB118, PCB138, PCB153, and PCB180, and individual standards of PCB169, and PCB143).

Various standard mixtures were prepared from these reagents according to analytical requirements. PCB 143 was used as the internal standard. After preparation, all standards were stored frozen at −20 °C and kept refrigerated at 4 °C during use.

### 2.3 Equipment

GC–MS analysis was conducted using a Shimadzu GC-2010 gas chromatograph equipped with an AOC-20s autosampler, an AOC-20i autoinjector, and a GC–MS-QP2010-Plus mass spectrometry detector, operated in negative chemical ionization (NCI) mode. Compound separation was achieved with an SLB 5MS (30 m x 0.25 mm x 0.25 μm) column from Supelco. Methane was the reaction gas, and helium was used as the carrier gas (Column flow: 0.89 mL/min; Flow control mode: linear velocity; Total flow: 21.6 mL/min). Injection volume was 1 μL in splitless mode, with a sampling time of 1 min. Injector temperature: 275 °C. Interface temperature: 300 °C. Detector temperature: 200 °C. The column temperature was maintained at 100 °C for 1 min, then increased to 200 °C at a rate of 60 °C/min, followed by a rise to 230 °C at a rate of 3 °C/min, then to 250 °C at a rate of 3 °C/min, and finally to 300 °C at a rate of 30 °C/min, where it was held for 10 min. Data analysis was performed using Shimadzu GC–MS Post-Run Analysis software.

Additional equipment used during sample preparation included a refrigerated centrifuge (Digicen 21R); a homogenizer (UltraTurrax IKA T18Basic with S18N-19G dispersing element) for homogenizing the liver samples, and a rotary shaker (IKA Trayster digital).

### 2.4 Method optimization

#### 2.4.1 Preliminary optimization of the QuEChERS extraction and cleanup process for chicken liver

For the method validation study, chicken livers (*Gallus gallus domesticus*) were purchased from a retail food chain for human consumption and refrigerated at 4 °C as a chlorinated-compound–free analytical matrix (blank liver, BL).

The extraction and purification process was based on the method developed by Norli et al. (34) for applying the QuEChERS technique to extract POPs from fatty salmon tissues. This method employs an ACN: THF (75:25) solvent mixture for extraction, followed by freezing the extract and subsequent exposure to CaCl₂ to remove lipids. The phases of this extraction and purification procedure are as follows:

- Homogenize 5 g of sample with 10 mL of Milli-Q water and 10 mL of solvent,
- Add a mixture of 4 g of MgSO₄, 1 g of NaCl, 0.5 g of disodium citrate, and 1 g of trisodium citrate; shake and centrifuge to achieve phase separation,
- Transfer 6 mL of the organic phase (solvent) to a test tube, freeze at −24 °C for 2 h, filter into a tube containing 1 g of CaCl₂, shake, and centrifuge again, and
- Finally, transfer the extract to a centrifuge tube containing 900 mg of MgSO₄ and 150 mg of PSA, shake, and centrifuge to obtain the final extract, which was then analyzed by gas chromatography.

##### 2.4.1.1 Extraction adjustments (water and solvent volumes)

Two strategies were tested on BL during the optimization process:

1. Decrease the amount of ultrapure water initially added (0, 3, 6.5, or 10 mL) to the 5 g liver sample during the homogenization process, and
2. Use two different extraction solvents for each homogenate with varying water proportions: 10 mL of ACN alone and 10 mL of an ACN: THF mixture in a 75:25 ratio.

Specifically, the procedure was applied as follows: 5 g aliquots of pre-homogenized sample were placed into 50 mL Falcon-type centrifuge tubes, to which the different chlorinated compound standards were added to obtain concentrations of 1, 10, 25, 50, and 100 ng/g. The samples were left refrigerated overnight. The following day, the internal standard PCB143 and the corresponding amounts of water (0, 3, 6.5, or 10 mL) were added to each sample, followed by 10 mL of either ACN or the ACN: THF (75:25) mixture, and vigorous manual shaking for 1 min. Subsequently, 4 g of MgSO₄, 1 g of NaCl, 0.5 g of disodium citrate, and 1 g of trisodium citrate dihydrate were added, and the samples were shaken on a rotary shaker for 10 min. The mixtures were then centrifuged at 4500 rpm for 5 min. The supernatant was transferred to a 15 mL Falcon-type centrifuge tube containing 1 g of CaCl₂, shaken, and centrifuged again at 4500 rpm for 5 min.

From the resulting supernatant, 1 mL was transferred to a 2 mL vial containing 300 mg of MgSO₄, 50 mg of PSA, and 50 mg of C18 [added to improve purification, although it had not been used in the method described by (34)]. After vigorous manual shaking for 1 min, the sample was centrifuged, and the supernatant was transferred to a 1 mL vial for chromatographic analysis.

##### 2.4.1.2 Sulfuric acid purification (clean-up)

Due to issues caused by co-extractive compounds (as explained in the Results and Discussion section), an alternative purification of the extracts using H₂SO₄ was performed. This method, routinely used in our laboratory and based on that described by Veierov and Aharonson (37), has the drawback that some chlorinated compounds (endosulfan, endrin, endrin aldehyde, and dieldrin) are destroyed by sulfuric acid and must therefore be excluded from analysis.

For this procedure, 5 g aliquots of blank liver (BL) were spiked with 75 μL of the internal standard (PCB 143) and 100 μL of each chlorinated compound standard mixture to obtain different liver concentrations (0, 1, 10, 25, 50, and 100 ng/g). The samples were left refrigerated overnight. The entire extraction process described previously was then repeated, using the different water proportions and both solvent systems (ACN and ACN: THF). After phase separation with the QuEChERS salts, each resulting extract was divided into two 5 mL aliquots:

- One aliquot was purified with PSA–C18, the solvent was evaporated under a nitrogen stream, reconstituted with 5 mL of hexane, and subsequently purified with H₂SO₄. This aliquot was used to assess whether PSA–C18 purification could improve the effectiveness of the H₂SO₄ cleanup,
- In the other aliquot, the solvent was evaporated under a nitrogen stream, reconstituted with 5 mL of hexane, and then purified directly with H₂SO₄.

In both cases, the hexane extract was evaporated to dryness and reconstituted with 200 μL of isooctane for chromatographic analysis.

#### 2.4.2 Final analytical method

Based on the assays described above, the final procedure was established as follows: sample preparation and extraction involved adding 75 μL of internal standard PCB143 and 10 mL of ACN to 5 g of minced liver. The mixture was then homogenized for 30 s using an UltraTurrax. Next, a salt mixture containing 4 g of MgSO₄, 1 g of NaCl, 0.5 g of disodium citrate, and 1 g of trisodium citrate dihydrate was added. The sample was shaken on a rotary shaker for 5 min, then centrifuged at 4000 rpm for 5 min at 4 °C.

Five milliliters of the supernatant were transferred to a 10 mL threaded glass tube, evaporated to dryness under a nitrogen stream, and then redissolved in 5 mL of hexane.

*Purification*: in the same 10 mL glass tube, 2 mL of H₂SO₄ was added dropwise, then the mixture was mixed on a rotary shaker. If the hexane extract was not completely clear, the H₂SO₄ was removed using a glass Pasteur pipette, and the step was repeated until the extract was clear. Once the hexane extract became fully transparent, it was transferred with a glass pipette to another glass tube and evaporated to dryness again under a nitrogen stream. The residue was then reconstituted with 200 μL of isooctane and immediately transferred into an insert placed inside a 2 mL vial for subsequent chromatographic analysis.

**Figure 1:**
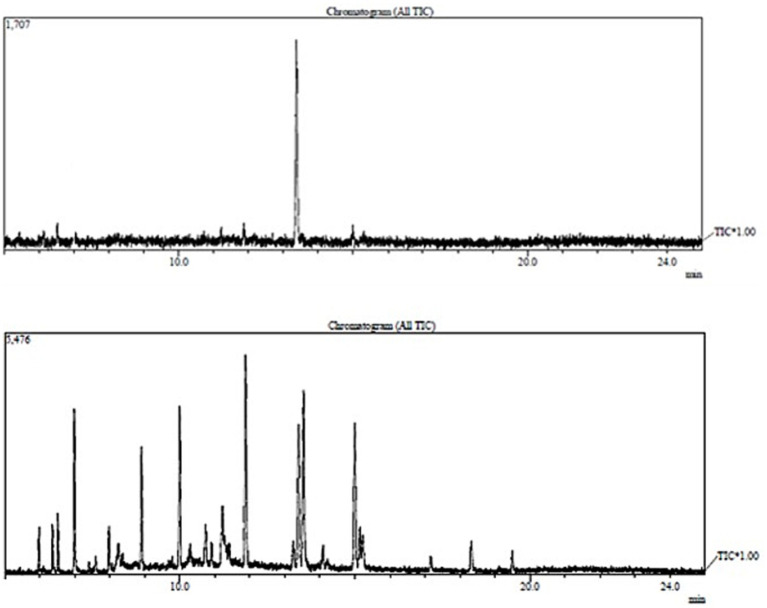
Chromatogram of commercial chicken liver (blank) extracts. Top, Blank spiked only with PCB143 (50 ng/g). Bottom, Blank spiked with PCB143 (50 ng/g) and a mixed standard of chlorinated compounds at 10 ng/g.

As shown in Figure 1, the blank liver was confirmed to be free of chlorinated compounds and therefore suitable for use in the method validation.

#### 2.4.3 Chromatographic analysis

Chromatographic analysis was performed under the conditions described above. The retention times for each compound are detailed in Table 1.

**Table 1:** Retention times for each compound.

Compound	Retention time (min)
Pesticides	
αHCH	5.99
βHCH	6.37
ɣHCH (lindane)	6.53
δHCH	7.01
Heptachlor	7.98
Aldrin	8.89
Heptachlor epoxide	9.98
DDE	11.91
DDD	13.58
Endosulfan sulfate	14.90
DDT	15.12
PCBs	
PCB28	7.63
PCB52	8.36
PCB101	10.89
PCB118	13.28
PCB153	14.12
PCB138	15.28
PCB169	19.48
PCB180	18.37

#### 2.4.4 Validation parameters of the analytical method

##### 2.4.4.1 Linearity

The linearity of an analytical method describes its capacity to produce results proportional to the analyte concentration within a specific range. The number of calibration points and the concentration range are chosen based on the method’s intended use.

To evaluate this parameter, calibration curves were created using BL samples fortified with pesticide standards at five different concentration levels (1, 10, 25, 50, and 100 ng/g), each analysed in triplicate, following the recommendations of the SANTE/12682/2019 guideline for analytical method validation (38). The regression equation was then determined for each compound.

The data were fit using the Pearson correlation coefficient, and the linearity criterion was set at r ≥ 0.9 (corresponding to the square root of the coefficient of determination, R^2^, for each calibration curve).

##### 2.4.4.2 Precision

Precision indicates the consistency of results when analyzing the same amount of a compound multiple times in the same sample under consistent conditions. It was assessed by measuring dispersion (coefficient of variation, CV, expressed as a percentage) among the results from three replicates for each chlorinated compound concentration in the BL samples. A precision value below 20% was set as the acceptance criterion, in line with the SANTE/12682/2019 guideline (38).

##### 2.4.4.3 Detection and quantification limits

The limit of detection (LOD) of the method was calculated as the analyte concentration (ng/g) corresponding to a signal-to-noise ratio of 3:1 in the blank sample. Similarly, the limit of quantification (LOQ) was defined as the concentration at which this ratio was 10:1. These values were determined using the GCMS Postrun Analysis software integrated into the chromatographic system, applied to the chromatogram corresponding to the lowest concentration detected in the linearity study.

#### 2.4.5 Final quantification method: internal standard with matrix calibration

The matrix effect (ME) for each pesticide was determined by comparing the calibration curves obtained from standards prepared in solvent and in BL (matrix) at known concentrations. The relationship between the slopes of the solvent-based and matrix-based calibration curves was calculated using the following equation (39):


$$\mathrm{ME}=\frac{\text{slope of matrix curve}}{\text{slope of solvent curve}}\times 100$$

Suppose the slopes of the solvent and matrix calibration curves are equal. In that case, no matrix effect is present, and the slope ratio equals 1, meaning the analyte signal in the matrix and in the solvent is identical. A matrix effect value greater than 100% indicates signal enhancement when the analyte is analyzed in the matrix. In contrast, a value below 100% indicates signal suppression compared with the analyte in the solvent.

According to the European Commission’s guideline SANTE/12682/2019 (2019) for the validation of analytical methods for pesticide residue determination in food and feed, an acceptable ME range is 100 ± 20%. Results outside this range can be corrected using an internal standard, as was done in this study, and are also acceptable as long as they do not negatively affect precision.

Therefore, in this study, an internal standard, PCB143, was added to all samples at a constant concentration of 50 ng/g. PCB143 was chosen because it shares the same chemical nature as the analytes of interest, does not affect the injection volume of the extract in the chromatograph, is not naturally found in the samples, elutes in the same chromatographic region as the other compounds, and does not react with the samples or analytes. This ensures it was analyzed under the same conditions as the target compounds.

For the BL samples used to generate calibration curves, the average peak area for PCB143 across all injections was calculated. This value was then used to determine a correction factor for the peak areas of all compounds in both BL and real samples, providing corrected areas for each one. Final quantification involved applying the corrected peak area of each compound to its corresponding calibration curve.

Using the internal standard quantification method, recovery studies were unnecessary because 100% recovery is assumed. This approach also avoids potential errors caused by changes in the final extract volume due to evaporation or partial loss, since the proportional relationship between the analyte and internal standard PCB143 concentrations remains constant throughout the extract.

### 2.5 Application of the final method

After optimizing the analytical method, it was used to analyze OCs and PCBs in 60 liver samples from three bird species that occupy different trophic levels: the white stork (*Ciconia ciconia*) as a secondary consumer, the red kite (*Milvus milvus*) as a tertiary consumer, and the griffon vulture (*Gyps fulvus*) as a scavenger. The birds were found dead in the natural environment of Extremadura. They were collected either by environmental officers from the Regional Government or by SEPRONA (the Nature Protection Service of the Spanish Civil Guard).

Afterward, the carcasses were sent to the Wildlife Rehabilitation Center “Los Hornos” in Sierra de Fuentes (Cáceres, Spain), where trained staff performed necropsies. When poisoning was suspected as a possible cause of death, the livers were removed and sent to our lab along with a Delivery Record that included all case-related details.

#### 2.5.1 Determination of liver fat content (lipids)

As an initial step before analyzing contaminants, the fat content of each liver sample, including the commercial chicken liver (BL), was measured gravimetrically (40). For this purpose, 1 g of liver was homogenized with 5 mL of petroleum ether and then centrifuged at 5000 rpm for 5 min. The supernatant (ether phase) was transferred to a pre-weighed aluminum tray and left at room temperature for 24 h. The solvent was then fully evaporated by heating at 105 °C for 1 h in an oven. After cooling to room temperature, the tray was weighed again to determine the amount of extracted fat.

#### 2.5.2 Descriptive and comparative statistical analysis

Due to high variability in results for each compound and species, and outliers, the median (Me) concentration was used instead of the arithmetic mean, as it provides a more reliable measure in these conditions. Statistical analyses were conducted using R® software. As the data were non-normally distributed, groups (species) were compared using the nonparametric Kruskal–Wallis test, and *p*-values < 0.05 were considered statistically significant. When the measured value was below the LOD (<LOD), a value equal to ½ LOD was used for statistical analysis.

## 3 Results

### 3.1 Method development

In the extracts from the two initial extraction strategies (using different water amounts and the two solvent types), no peaks of chlorinated compounds were detected at the spiked concentration of 50 ng/g or lower. After concentration to 100 μL, detector saturation occurred due to insufficient cleanup of co-extracted compounds.

After adding an extra purification step with H₂SO₄ washing, chromatographic analysis of both extract types showed no significant differences in extraction efficiency, whether water was added or not, or whether ACN alone or the ACN: THF mixture was used as the solvent. Therefore, extraction was simplified by omitting water and using ACN as the sole solvent.

No differences were observed regarding the inclusion or omission of the QuEChERS purification step (PSA + C18), and the issue of co-extractive compounds was resolved after the H₂SO₄ wash. Additionally, this method allowed the extract to be concentrated 25 times, improving the detection of chlorinated compounds at lower concentrations. Therefore, the most cost-effective option—direct purification with H₂SO₄ followed by extract concentration—was ultimately chosen.

### 3.2 Final method validation

#### 3.2.1 Linearity and precision

After triplicate analysis of each BL sample fortified with pesticide standards at five concentration levels (1, 10, 25, 50, and 100 ng/g), the calibration equation for each compound was determined. Compliance with the linearity acceptance criterion (correlation coefficient *r* ≥ 0.9) is detailed in Table 2, along with the precision values, which also meet the acceptance criterion of CV < 20%.

**Table 2:** Linearity values (r) and precision (coefficient of variation, CV %) were determined from triplicate analysis of each standard concentration (range: 1–100 ng/g) in blank liver samples.

Compound	Linearity (r)	CV (%) 1 ng/g	CV (%) 10 ng/g	CV (%) 25 ng/g	CV (%) 50 ng/g	CV (%) 100 ng/g	LOD (ng/g)	LOQ (ng/g)	ME (%)
αHCH	0.99	14.65	3.75	12.05	3.61	1.62	0.79	2.63	65.45
βHCH	0.99	10.22	3.50	8.22	15.80	11.61	1.95	6.50	138.28
ɣHCH	0.98	7.99	4.30	8.43	10.30	15.22	1.50	5.00	113.50
δHCH	0.99	6.35	2.08	3.56	14.01	5.92	0.86	2.87	172.80
Heptachlor	0.99	5.25	9.82	5.35	6.34	9.43	0.49	1.62	95.71
Aldrin	0.99	19.35	5.98	4.03	13.93	9.85	2.03	6.78	98.68
Heptachlor epoxide	0.99	8.40	15.15	7.40	5.80	4.04	1.22	4.05	110.49
DDE	0.99	7.16	3.34	3.02	5.39	5.86	0.20	0.67	163.23
DDD	0.99	6.31	1.25	6.55	1.07	1.77	0.80	2.68	105.47
Endosulfan sulfate	0.97	6.64	2.66	5.18	5.50	3.21	0.62	2.05	134.16
DDT	0.98	1.55	4.41	3.03	7.64	1.62	0.43	1.42	159.30
PCB28	0.98	—	9.61	3.23	6.72	7.22	0.93	3.11	107.74
PCB52	0.99	—	2.42	8.03	13.19	4.34	1.50	4.99	119.16
PCB101	0.99	13.92	11.51	9.48	4.43	2.99	4.49	14.97	142.00
PCB118	0.99	—	10.17	2.59	0.80	3.32	1.52	5.08	135.28
PCB138	0.99	10.93	5.23	9.16	5.16	4.17	1.72	5.74	139.96
PCB153	0.99	7.65	8.76	3.62	2.72	2.42	2.75	9.18	123.77
PCB169	0.99	—	5.11	7.27	1.53	0.63	3.89	12.97	160.89
PCB180	0.99	—	1.61	1.91	1.05	5.01	1.01	3.38	136.12

LOD, LOQ, and matrix effect (ME) were calculated for each compound.

#### 3.2.2 Limit of detection (LOD) and limit of quantification (LOQ)

The LOD and LOQ values obtained for each compound are presented in Table 2.

#### 3.2.3 Matrix effect (ME) determination

The matrix effect (ME) for each pesticide was determined (Table 2), with values calculated from the calibration curves of standards, spiked at known concentrations in solvent and in BL (matrix).

The instrumental response increased for all analytes due to the matrix effect, except for *α*-HCH, heptachlor, and aldrin.

### 3.3 Analysis of OCs and PCBs in wild liver

It is important to note that one of the main effects of sulfuric acid purification was the destruction of certain chlorinated pesticides (α and *β*-endosulfan, dieldrin, endrin, endrin aldehyde, and chlordane), which could not be analyzed, therefore (41).

After method optimization and validation, lipid content was measured in commercial chicken liver (blank) and wild bird livers. The commercial liver averaged 3.20% lipids, while wild bird samples showed lower values (detailed results are shown in Table 3). Subsequently, OC and PCB residues were identified and quantified in the liver samples. The analyzed compounds included 19 POPs: the organochlorine pesticides *α*-HCH, β-HCH, *γ*-HCH, *δ*-HCH, heptachlor, aldrin, heptachlor epoxide, DDE, DDD, endosulfan sulfate (a metabolite of endosulfan), and DDT, as well as PCBs 28, 52, 101, 118, 153, 138, 180, and 169 (detailed results are shown in Table 4 and Supplementary Table S1).

**Table 3:** Lipid content (%) in wild bird liver samples: white stork, red kite, and griffon vulture.

Parameter	White stork *Ciconia ciconia*	Red kite *Milvus milvus*	Griffon vulture *Gyps fulvus*
Sample size (n)	20	20	20
Range	0.34–1.84	1.24–2.36	0.60–3.01
Mean (%)	1.19	1.68	1.47
Standard error of the mean	0.11	0.07	0.14
Median	1.12	1.63	1.52
Coefficient of variation (%)	42.72	19.38	42.84

**Table 4:** Concentration ranges (ng/g) for each bird species for chlorinated compounds with detection frequencies above the LOD (>LOD) in less than 50% of samples.

Compound	Species	Range (ng/g)	>LOD (%)
δHCH	White stork *Ciconia ciconia*	<LOD—1.70	10.00
δHCH	Red kite *Milvus milvus*	<LOD—19.86	30.00
δHCH	Griffon vulture *Gyps fulvus*	<LOD—64.71	35.00
Heptachlor	White stork *Ciconia ciconia*	<LOD—2.31	15.00
Heptachlor	Red kite *Milvus milvus*	<LOD—16.62	30.00
Heptachlor	Griffon vulture *Gyps fulvus*	<LOD—1.49	10.00
Aldrin	White stork *Ciconia ciconia*	<LOD—260.43	20.00
Aldrin	Red kite *Milvus milvus*	<LOD—48.04	5.00
Aldrin	Griffon vulture *Gyps fulvus*	<LOD—59.10	20.00
Endosulfan sulfate	White stork *Ciconia ciconia*	<LOD—12.89	30.00
Endosulfan sulfate	Red kite *Milvus milvus*	<LOD—8.18	30.00
Endosulfan sulfate	Griffon vulture *Gyps fulvus*	<LOD—0.83	10.00
DDT	White stork *Ciconia ciconia*	<LOD—4.87	10.00
DDT	Red kite *Milvus milvus*	<LOD—52.71	40.00
DDT	Griffon vulture *Gyps fulvus*	<LOD—45.85	25.00
PCB52	White stork *Ciconia ciconia*	<LOD—10.62	20.00
PCB52	Red kite *Milvus milvus*	<LOD—15.69	35.00
PCB52	Griffon vulture *Gyps fulvus*	<LOD—10.19	25.00
PCB169	White stork *Ciconia ciconia*	<LOD—15.18	25.00
PCB169	Red kite *Milvus milvus*	<LOD—6.39	25.00
PCB169	Griffon vulture *Gyps fulvus*	<LOD—10.91	10.00

**Figure 2:**
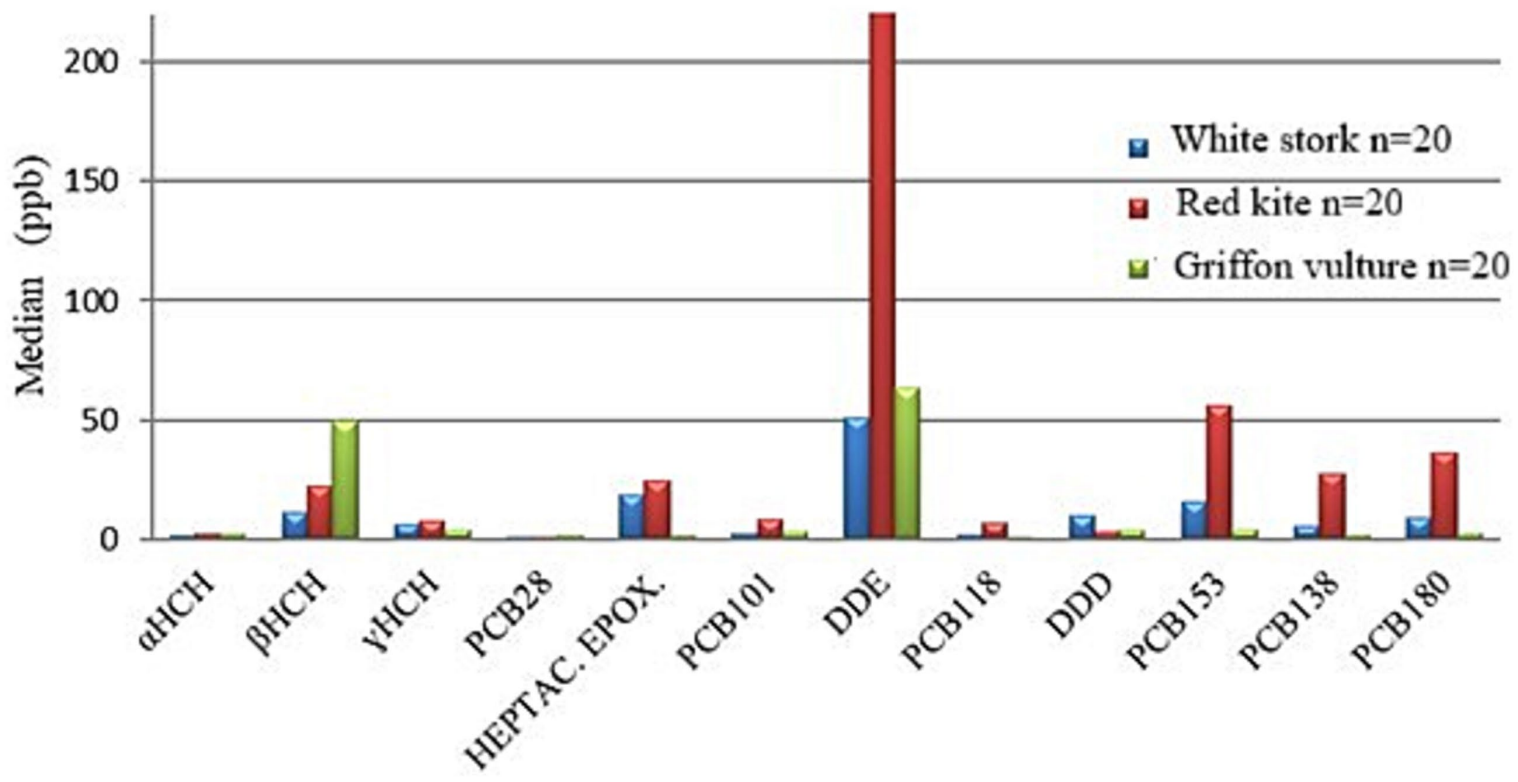
Median (ng/g) concentrations of each compound measured for each species.

Due to high data dispersion, medians were used and analyzed with nonparametric tests (Figure 2). Significant interspecies differences were observed for eight compounds, as shown in Table 5 (a *p* < 0.05 and b *p* < 0.05). These differences were identified between the concentrations of DDE, DDD, PCB153, PCB138, and PCB180 in white storks and red kites; between α-HCH, heptachlor epoxide, and PCB153 in white storks and griffon vultures; and between heptachlor epoxide, DDE, PCB118, PCB153, PCB138, and PCB180 in red kites and griffon vultures.

**Table 5:** Mean and range of results obtained in this study^1^, compared with the ranges reported for red kite and griffon vulture liver by Van Drooge et al. (61)^2^ and Hela et al. (51)^3^.

Compound	White stork *Ciconia ciconia*	Red kite *Milvus milvus*	Griffon vulture *Gyps fulvus*
αHCH	2.50 [< LOD–9.18]1	6.90 [< LOD–68.12]^1^	9.41 [< LOD–69.17]^1^ [<LOD–17.4]^3^
βHCH	42.28 [< LOD–215.31]^1^	62.26 [2.00–269.75]^1^ [<LOD–26.1]^2^	264.05 [< LOD–2199.79]^1^ [<LOD–33.7]^2^ [<LOD–3.95]^3^
δHCH	[< LOD–1.70]^1^	[< LOD–19.86]^1^	[< LOD–64.71]^1^ [<LOD–7.08]^3^
ɣHCH	6.18 [< LOD–16.87]^1^	9.89 [2.00–25.74]^1^ [<LOD–42.5]^2^	5.62 [< LOD–22.86]^1^ [<LOD–51.9]^2^ [<LOD–2.37]^3^
Heptachlor epoxide	271.36 [< LOD–3166.46]^1^	34.94 [< LOD–174.59]^1^	9.80 [< LOD–89.00]^1^ [Not detected]^3^
Heptachlor	[< LOD–2.31]^1^	[< LOD–16,62]^1^ [<LOD–17.3]^2^	[< LOD—1.49]^1^ [<LOD-46.3]^2^ [<LOD-28.6]^3^
Endosulfan sulfate	[< LOD–12.89]^1^	[< LOD–8.18]^1^	[< LOD–0.83]^1^ [Not detected]^3^
Aldrin	[< LOD–260.43]^1^	[< LOD–48.04]^1^	[< LOD–59.10]^1^ [Not detected]^3^
DDE	200.56 [6.17–845.33]^1^	314.21 [31.12–954.79]^1^ [228–1,528]^2^	169.44 [5.30—557.74]^1^ [16–2,285]^2^ [170.31–191.06]^3^
DDD	13.25 [1.00–58.60]^1^	4.45 [< LOD–14.37]^1^	27.02 [< LOD–161.40]^1^ [3.42–19.37]^3^
DDT	[< LOD–4.87]^1^	[< LOD–52.71]^1^	[< LOD–45.85]^1^ [9.70–12.89]^3^
PCB28	4.84 [< LOD-41.26]^1^	2.77 [< LOD–14.08]^1^	3.60 [< LOD–27.66]^1^ [0.43–67.60]^3^
PCB52	[< LOD–10.62]^1^	[< LOD–15.69]^1^	[< LOD–10.19]^1^ [0.37–2.68]^3^
PCB101	14.54 [< LOD–66.86]^1^	15.16 [< LOD–78.06]^1^	17.86 [< LOD–86.49]^1^ [<LOD–2.80]^3^
PCB118	18.70 [< LOD-201.31]^1^	10.95 [< LOD–66.28]^1^	9.22 [< LOD–86.42]^1^ [<LOD–69.50]^3^
PCB138	50.40 [< LOD–550.81]^1^	35.53 [4.67–146.33]^1^	24.30 [< LOD–189.31]^1^ [<LOD–3.90]^3^
PCB153	113.32 [2.67–1088.25]^1^	81.70 [< LOD–389.85]^1^	67.88 [< LOD–681.99]^1^ [9.36–22.70]^3^
PCB169	[< LOD–15.18]^1^	[< LOD–6.39]^1^	[< LOD–10.91]^1^
PCB180	82.40 [< LOD–827.84]^1^	48.70 [5.99–204.80]^1^	52.24 [< LOD–463.30]^1^ [<LOD–9.09]^3^

1 = data in this study, 2 = data from Van Drooge et al. (61), and 3 = Data from Hela et al. (51).

The median values for each compound and species (Figure 2) indicate that DDE had the highest concentration in red kites. It was also detected, to a lesser extent, in all griffon vulture and in all white stork samples. DDE was the only compound found in 100% of the analyzed samples. DDD, also a DDT metabolite, had a detection frequency of 90%. The second most frequently detected compound was *β*-HCH (92%), which is also among the most resistant chlorinated compounds within the HCH group to both biotic and abiotic degradation processes. α-HCH was found in 88% of the samples, and δ-HCH (lindane) in 73%. The average detection frequency of compounds with values >LOD was higher in red kite liver samples (85.42%) and white storks (81.25%) than in griffon vultures (69.58%). Similarly, red kites showed higher median concentration values (35.22 ng/g) compared to vultures (12.04 ng/g) and storks (11.08 ng/g), although the mean concentration was slightly lower (52.29 ng/g) than in vultures (55.04 ng/g) and storks (68.36 ng/g). Data dispersion (CV) was lower in red kites (119.64%) than in storks (174.61%) and vultures (187.34%), due to the presence of extreme values in the latter two species (2199.79 ng/g of β-HCH in one vulture, 3166.46 ng/g of heptachlor epoxide in one stork, and 1088.25 ng/g of PCB153 in another stork).

The sum of PCB median concentrations was 37.95 ng/g for white storks, 137.87 ng/g for red kites, and 17.47 ng/g for griffon vultures. And, specifically, detection frequency increased with the number of chlorine atoms, following the order: PCB180 (88%) > PCB153 (83%) > PCB138 (80%) > PCB118 (63%) > PCB101 (57%) > PCB28 (52%).

In seven of the analyzed compounds, concentrations below the LOD were observed in more than 50% of the samples (detection frequency). As a result, these compounds were excluded from the statistical analysis (see Table 4 for detailed results). The detection frequency of these seven compounds at concentrations above the LOD was low, ranging from 5% to 40%. The least frequently detected compound in the study was aldrin, which was found in only 1 of the 20 red kite samples analyzed.

Across all samples, an average of 11 distinct chlorinated compounds (values > LOD) were detected per liver.

## 4 Discussion

The variability observed among species is consistent with previous reports in European birds, supporting the identified exposure patterns. Studies by Tomza-Marciniak et al. (42) and Naso et al. (43) have described comparable concentration ranges for many of the compounds analyzed. In the latter study, no significant differences in organochlorine pesticide levels were found among species or trophic groups, although trends were observed, including higher DDE concentrations in carnivorous species and elevated PCB levels in omnivorous, opportunistic, and carnivorous species.

In the present study, although red kites (*Milvus milvus*) generally exhibited higher contamination levels than griffon vultures and white storks, no clear trophic-level-related accumulation pattern was identified. This suggests that diet alone does not fully explain POP accumulation and points to the contribution of additional biological factors. Within this framework, metabolic capacity appears to be a key determinant. Birds generally exhibit limited ability to metabolize POPs, leading to faster and greater accumulation under comparable exposure conditions (44). This capacity also varies among species as a function of mixed-function oxidase (MFO) enzyme activity, which is influenced by feeding habits (45).

Omnivorous and opportunistic species exhibit higher MFO activity, enabling more flexible enzymatic responses and, potentially lower accumulation of certain compounds. In contrast, predatory species show lower enzyme activity and a greater tendency to accumulate organochlorine compounds. Accordingly, the reported correlation between the omnivory index and detoxification enzyme activity (46) may partly explain the interspecific differences observed here, such as lower levels in white storks than in red kites.

Taken together, these findings indicate that trophic level alone does not determine POP accumulation and should be interpreted within a broader physiological and ecological framework.

Beyond interspecific variability, the results reveal clear patterns associated with the physicochemical properties of the analyzed compounds. In particular, the predominance of DDE across all species, in both frequency and concentration, confirms its high stability and resistance to degradation, which promote its accumulation in the environment and in biological tissues. This pattern has been widely reported in birds (17, 43, 47–54), reinforcing its role as one of the most relevant contaminants in biomonitoring studies.

Similarly, among PCBs, the predominance of highly chlorinated congeners such as PCB153, PCB138, PCB118, and PCB180 reflects their greater bioaccumulation potential compared with less chlorinated congeners, which are more susceptible to metabolic transformation and excretion by organisms (43, 55–60), in agreement with previous findings (17, 42, 43, 51). This behavior is closely related to their molecular structure, which limits metabolism and elimination, promoting tissue accumulation (42, 43, 61).

For HCH isomers, the predominance of *β*-HCH, both in concentration and detection frequency, can be attributed to its greater stability against degradation (17, 49). In contrast, the relatively high levels of *γ*-HCH (lindane) suggest relatively recent exposure at elevated levels, despite its faster metabolism and excretion (60) and existing regulatory restrictions. Similar findings have been reported previously, although with variability in the detected levels (17, 49). By contrast, Naso et al. (43) did not detect γ-HCH in liver samples from bird species with different feeding habits, highlighting inter-study variability and possible differences in exposure sources. In feather-based studies, *α*, β, and γ-HCH isomers were detected in all samples, although at lower concentrations than those observed here, a pattern also reported for DDTs and PCBs Dahmardeh (62).

Furthermore, the occurrence of extreme values of PCB153 and β-HCH in some samples reflects their high resistance to biotic and abiotic degradation (56).

These results indicate that POP distribution patterns are not solely determined by biological or ecological factors but are strongly influenced by the physicochemical properties of each compound, which govern their persistence, bioaccumulation, and environmental behavior.

Comparisons among studies should be interpreted with caution and regarded as indicative rather than conclusive. This is due to the intra and interspecific differences discussed previously, as well as variations in statistical methods, sample characteristics, and environmental conditions at collection sites.

Existing studies show high variability, as highlighted by Naso et al. (43) across multiple species and regions. Although many studies report that trophic level and species-specific metabolism influence POP accumulation (17, 45, 63–65), others indicate that individual factors such as sex, age, and body condition exert a greater influence on POP accumulation (50, 66–71).

Residue levels also vary seasonally and correlate with body condition in birds: lower residue levels are observed during periods of food abundance (and therefore better body condition), whereas higher concentrations are detected during periods of food scarcity and poorer condition (68, 72–74).

Wienburg and Shore (75) analyzed, over a five-year period across the United Kingdom, the intra and interspecific factors influencing PCB accumulation in three species from different trophic levels: the Eurasian sparrowhawk (*Accipiter nisus*), the common kestrel (*Falco tinnunculus*), and the grey heron (*Ardea cinerea*). Their study showed that body condition was the main determinant of PCB accumulation, whereas age, sex, and time of death had smaller effects. Collectively, these factors had a greater influence than species, metabolic capacity, and geographic exposure. This study reinforces the idea that individual factors may have a greater influence on POP accumulation than species or trophic level.

This variability underscores the need for more representative, less biased data. In this context, non-invasive samples such as feathers represent valuable alternatives to liver or muscle. The accumulation of organochlorine compounds in feathers has been increasingly studied (76–79), and correlations with concentrations detected in liver and muscle have been reported (80).

The availability of effective and accessible methods for analyzing POPs in complex biological matrices remains a critical aspect of environmental biomonitoring, particularly in laboratories with limited analytical instrumentation. In this context, the QuEChERS methodology represents a promising option due to its operational advantages of versatility, cost, and simplicity. The method is increasingly used for pesticide analysis in animal tissues and has shown satisfactory recovery rates (81). In recent years, several studies have applied it to the analysis of POPs in different animal tissues, including salmon and mussel meat (82), raptor and camel blood or serum (16, 32, 85), bovine, ovine, and porcine liver (36), as well as hedgehog and bat liver (35).

Our results indicate that the QuEChERS extraction process is suitable for liver samples. However, the purification step is insufficient for chromatographic analysis of very low concentrations (<100 ng/g) in laboratories using single-quadrupole GC–MS systems. In this regard, the adaptation of the QuEChERS method developed and validated in this study proved effective for POP extraction and purification in avian liver using GC–MS. Although the procedure was not faster or simpler than the original method due to the use of sulfuric acid during the purification step, it enabled the effective analysis of 19 POPs in a complex biological matrix. The method was applied to liver samples from 60 birds of three species from different trophic levels collected in Extremadura (Spain), with an average of 11 compounds detected (>LOD) per sample.

Collectively, the results show that POPs are widely present across the analyzed species, indicating continuous environmental exposure. The high prevalence of compounds such as DDE and PCBs reflects an ongoing legacy of historical contamination, consistent with their persistence and resistance to degradation. The higher detection frequency and concentrations of more persistent compounds, compared with less chlorinated congeners, further reflect differences in metabolic behavior and environmental stability.

These findings reinforce the continued relevance of POPs as an environmental concern and underscore the need for sustained wildlife biomonitoring, highlighting the role of raptors as sentinel species within the One Health framework. In this regard, liver contamination in raptors not only reflects individual pollutant accumulation but also integrates ecological processes such as biomagnification and anthropogenic pressure, reinforcing their value as sentinels of environmental and potential human health risks.

In this context, robust analytical methods are essential for biomonitoring in complex matrices. The extraction and clean-up approach developed and validated in this study, based on QuEChERS, proved effective for the analysis of multiple POPs in avian liver using single-quadrupole GC–MS. Although the use of sulfuric acid in the clean-up step complicates the original method, its applicability to lipid-rich matrices supports its use as an accessible analytical tool for environmental monitoring in laboratories without advanced tandem mass spectrometry instrumentation.

Further efforts are needed to increase sample size, reduce sampling variability, and collect more detailed exposure and individual data. Such efforts will contribute to a better understanding of contaminant accumulation dynamics, improve wildlife trend assessment, and facilitate more robust cross-study comparisons.

## Data Availability

The raw data supporting the conclusions of this article will be made available by the authors, without undue reservation.
