# Supplementary material for: Modified QuEChERS methodology for the analysis of OCs and PCBs in liver and its application in wild birds (white stork, red kite, and griffon vulture)
Source: Front Vet Sci. 2026 Jun 12;13:1843311. doi: 10.3389/fvets.2026.1843311 (PMC13303352; doi:10.3389/fvets.2026.1843311)
Supplement: Supplementary file 1 [file Table_1.docx]

Supplementary Material

# Supplementary Table

Supplementary Table 1. Descriptive statistics (median, mean, and its standard error, concentration range, and coefficient of variation—CV) were obtained for each bird species studied for those chlorinated compounds with detection frequencies above 50% of values greater than the LOD (>LOD). Superscripts indicate pairs of species for which the statistical analysis revealed significant differences.

| Compound | Parameter | White stork  *Ciconia ciconia*  (n = 20) | Red kite  *Milvus milvus*  (n = 20) | Griffon vulture  *Gyps fulvus*  (n = 20) |
| --- | --- | --- | --- | --- |
| **αHCH** | Median (ng/g) | 1.40ᵇ | 2.35 | 2.24 |
|  | Mean ± SD (ng/g) | 2.50 ± 0.54 | 6.90 ± 3.31 | 9.41 ± 3.82 |
|  | Range (ng/g) | <LOD - 9.18 | <LOD - 68.12 | <LOD - 69.17 |
|  | CV (%) | 95.91 | 214.87 | 181.49 |
|  | >LOD (%) | 80 | 90 | 95 |
| βHCH | Median (ng/g) | 10.99 | 22.53 | 49.63 |
|  | Mean ± SD (ng/g) | 42.28 ± 14.53 | 62.26 ± 17.35 | 264.05 ± 133.49 |
|  | Range (ng/g) | <LOD - 215.31 | 2.00-269.75 | <LOD - 2199.79 |
|  | CV (%) | 153.66 | 124.63 | 226.09 |
|  | >LOD (%) | 80 | 100 | 95 |
| ɣHCH | Median (ng/g) | 6.07 | 7.89 | 3.71 |
|  | Mean ± SD (ng/g) | 6.18 ± 0.98 | 9.89 ± 1.97 | 5.62 ± 1.35 |
|  | Range (ng/g) | <LOD - 16.87 | <LOD - 25.74 | <LOD - 22.86 |
|  | CV (%) | 70.79 | 89.26 | 107.97 |
|  | >LOD (%) | 80 | 75 | 65 |
| Heptachlor epoxide | Median (ng/g) | 18.29 ^b^ | 24.45 ^b^ | 2.58 |
|  | Mean ± SD (ng/g) | 271.36 ± 166.30 | 34.94 ± 10.37 | 9.80 ± 4.54 |
|  | Range (ng/g) | <LOD - 3166.46 | <LOD - 174.59 | <LOD - 89.00 |
|  | CV (%) | 274.06 | 132.78 | 207.26 |
|  | >LOD (%) | 90 | 85 | 60 |
| DDE | Median (ng/g) | 51.13 ^a^ | 227.00 ^b^ | 63.20 |
|  | Mean ± SD (ng/g) | 200.56 ± 60.02 | 314.21 ± 66.46 | 169.44 ± 44.39 |
|  | Range (ng/g) | 6.17-845.33 | 31.12-954.79 | 5.30-557.74 |
|  | CV (%) | 133.84 | 94.59 | 117.17 |
|  | >LOD (%) | 100 | 100 | 100 |
| DDD | Median (ng/g) | 9.85 ^a^ | 3.19 | 3.74 |
|  | Mean ± SD (ng/g) | 13.25 ± 3.07 | 4.45 ± 0.85 | 27.02 ± 11.18 |
|  | Range (ng/g) | 1.00 - 58.60 | <LOD - 14.37 | <LOD - 161.40 |
|  | CV (%) | 103.75 | 84.98 | 210.18 |
|  | >LOD (%) | 100 | 90 | 80 |
| PCB28 | Median (ng/g) | 0.77 | 0.75 | 1.96 |
|  | Mean ± SD (ng/g) | 4.84 ± 2.08 | 2.77 ± 0.88 | 3.60 ± 1.41 |
|  | Range (ng/g) | <LOD - 41.26 | <LOD - 14.08 | <LOD - 27.66 |
|  | CV (%) | 192.30 | 141.38 | 176.49 |
|  | >LOD (%) | 50 | 50 | 55 |
| PCB101 | Median (ng/g) | 2.25 | 8.15 | 4.34 |
|  | Mean ± SD (ng/g) | 14.54 ± 3.51 | 15.16 ± 4.09 | 17.86 ± 5.27 |
|  | Range (ng/g) | <LOD - 66.86 | <LOD - 78.06 | <LOD - 86.49 |
|  | CV (%) | 107.99 | 120.63 | 131.95 |
|  | >LOD (%) | 50 | 70 | 50 |
| PCB118 | Median (ng/g) | 2.07 | 6.98 ^b^ | 4.41 |
|  | Mean ± SD (ng/g) | 18.70 ± 10.18 | 10.95 ± 3.10 | 9.22 ± 4.33 |
|  | Range (ng/g) | <LOD - 201.31 | <LOD - 66.28 | <LOD - 86.42 |
|  | CV (%) | 243.55 | 126.64 | 210.18 |
|  | >LOD (%) | 70 | 70 | 50 |
| PCB138 | Median (ng/g) | 5.66 ^a^ | 27.28 ^b^ | 1.87 |
|  | Mean ± SD (ng/g) | 50.40 ± 28.95 | 35.53 ± 20.12 | 24.30 ± 11.21 |
|  | Range (ng/g) | <LOD - 550.81 | 4.67 - 146.33 | <LOD - 189.31 |
|  | CV (%) | 256.85 | 93.38 | 206.44 |
|  | >LOD (%) | 80 | 100 | 60 |
| PCB153 | Median (ng/g) | 15.56 ^a,b^ | 55.97 ^b^ | 4.13 |
|  | Mean ± SD (ng/g) | 113.32 ± 57.33 | 81.70 ± 20.12 | 67.88 ± 36.26 |
|  | Range (ng/g) | 2.67 - 1088.25 | <LOD - 389.85 | <LOD - 681.99 |
|  | CV (%) | 226.26 | 110.15 | 238.92 |
|  | >LOD (%) | 100 | 95 | 55 |
| PCB180 | Median (ng/g) | 8.94 ^a^ | 36.04 ^b^ | 2.66 |
|  | Mean ± SD (ng/g) | 82.40 ± 43.36 | 48.70 ± 11.15 | 52.24 ± 27.33 |
|  | Range (ng/g) | <LOD – 827.84 | 5.99 - 204.80 | <LOD - 463.30 |
|  | CV (%) | 236.36 | 102.34 | 233.94 |
|  | >LOD (%) | 95 | 100 | 70 |

^a^ (*p*<0.05 *vs.* Red kite); ^b^ (*p*<0.05 *vs.* Griffon vulture)
